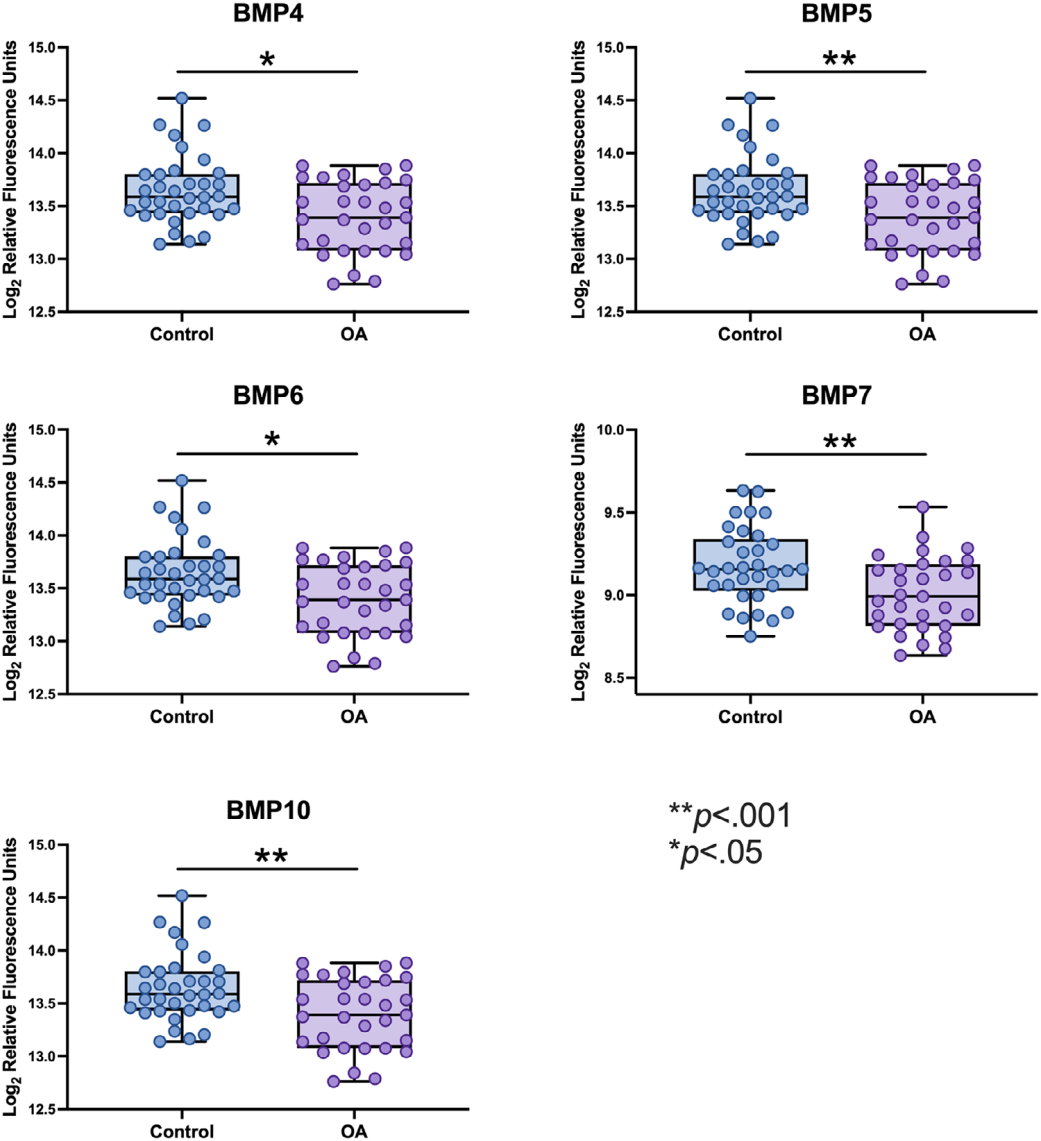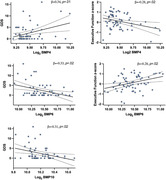# Bone morphogenetic protein levels relate to osteoarthritis and neurobehavioral outcomes in cognitively unimpaired older adults

**DOI:** 10.1002/alz.090089

**Published:** 2025-01-09

**Authors:** Valentina E. Diaz, Emily W. Paolillo, Coty Chen, Anna M. VandeBunte, Claire J. Cadwallader, Charles A. Schurman, Birgit Schiling, Bruce L. Miller, Joel H. Kramer, Kaitlin B. Casaletto, Rowan Saloner

**Affiliations:** ^1^ Memory and Aging Center, UCSF Weill Institute for Neurosciences, University of California, San Francisco, San Francisco, CA USA; ^2^ Buck Institute for Research on Aging, Novato, CA USA

## Abstract

**Background:**

Osteoarthritis (OA) is a degenerative disorder of bone aging and risk factor for cognitive decline. Bone morphogenetic proteins (BMPs) are growth factor proteins that regulate skeletal and neural development, and circulating BMPs may mediate molecular cross‐talk between bone and brain. The present study examined plasma BMP levels in relation to OA and neurobehavioral outcomes in cognitively unimpaired (CU) older adults.

**Methods:**

Participants included 65 CU adults (mean age= 76.45, 60% female) enrolled in the UCSF Brain Aging Network for Cognitive Health (BrANCH) study who underwent cross‐sectional neurological examination, neuropsychological testing, and blood draw. Participants were classified as OA (n=31) or control (CTL; n=34) based on clinician exam (UDS Medical Conditions). Plasma was assayed for 11 different BMPs via SomaScan v4.1. Composite z‐scores were calculated for cognitive domains of memory and executive functioning. Mood symptoms were quantified with the 30‐item Geriatric Depression Scale (GDS). ANCOVA compared BMP concentrations between OA and CTL, adjusting for age and sex. Separate multiple linear regressions examined the relationship between OA‐associated BMPs and neurobehavioral outcomes across the entire sample (cognitive z‐scores, GDS total score), adjusting for age, sex, and education.

**Results:**

OA and CTL groups did not significantly differ on demographics or neurobehavioral outcomes (ps>0.10). Five BMPs were differentially abundant (ps<.05) between OA and CTL (lower in OA: BMP5, BMP7, BMP10, BMP6; higher in OA: BMP4). In neurobehavioral models, lower BMP6 and higher BMP4 levels related to worse executive functioning (BMP6: β=0.27, p=.03; BMP4: β=‐0.28, p=.02) and higher GDS scores (BMP6: β=‐0.33, p=.02; BMP4: β=0.34, p=.01). Higher BMP10 also related to lower GDS scores (β=‐0.31, p=.02). No associations were observed between BMPs and memory z‐scores (ps>0.05).

**Conclusion:**

In a cohort of CU older adults, OA related to differential plasma abundance of BMPs. In turn, circulating BMP levels, specifically BMP4, BMP6, and BMP10, were associated with executive function and depressive symptoms. These data help provide a molecular link connecting skeletal disorders of aging (e.g., OA) to adverse neurobehavioral outcomes. Given the diverse immunovascular, metabolic, and growth regulatory functions of BMPs, additional mechanistic work is needed to identify the precise pathways by which systemic BMPs interact with the central nervous system.